# Zero-Shot 3D Object Classification via Graph-Based Local Geometric Features and Depth-Aware Multi-View Projection

**DOI:** 10.3390/s26144598

**Published:** 2026-07-20

**Authors:** Wenchao He, Ying Liu, Hongxi Zhao, Yiran Shi

**Affiliations:** 1 Department of Electromechanical and Information Engineering, Changchun Humanities and Sciences College, Changchun 130117, China; 2 College of Communication Engineering, Jilin University, Changchun 130015, China; 3 College of Electronic Science and Engineering, Jilin University, Changchun 130015, China

**Keywords:** point cloud, zero-shot classification, graph neural network, multi-view projection, geometric features, CLIP

## Abstract

Three-dimensional sensing technologies can rapidly acquire 3D point cloud data for object perception and scene understanding. However, point cloud-based object classification is still constrained by limited labeled data and high computational complexity. At present, feature extractors pretrained on large-scale 2D datasets have achieved strong performance in zero-shot 2D classification. Therefore, projecting 3D point clouds into 2D images and leveraging well-established 2D pretrained models for zero-shot point cloud classification has become an effective strategy. In this strategy, generating high-fidelity 2D projections is a critical challenge. To address this challenge, this paper proposes a zero-shot 3D object classification framework based on a multi-scale local geometric feature extraction module and depth-aware multi-view projection. Specifically, point clouds are first modeled as graph structures. Multi-scale radii are used to adjust the receptive field during feature extraction, thereby capturing both fine-grained and large-scale local geometric features. Multi-view 2D images are then generated through depth-wise feature accumulation. These images are fed into a frozen CLIP model for zero-shot classification. The proposed method preserves the structural characteristics of point clouds and reduces the domain gap between point clouds and 2D images. Experiments are conducted on the ModelNet10, ModelNet40, and ScanObjectNN datasets. The results show that the proposed method outperforms current mainstream zero-shot 3D point cloud classification methods. These results suggest that the proposed framework offers an effective solution for point cloud object classification in complex scenarios.

## 1. Introduction

Three-dimensional point clouds are a fundamental data modality in autonomous driving, intelligent surveillance, remote sensing, and robotic perception. With the widespread adoption of 3D sensing devices, the scale of unlabeled point cloud data has been rapidly increasing. However, manual annotation is costly, and the categories in open environments change frequently. Traditional supervised learning methods struggle to cover unseen categories. Therefore, zero-shot 3D point cloud classification, which recognizes unseen classes without class-specific training samples, has become an important research problem.

Mahalanobis Existing point cloud recognition methods can be broadly categorized into point-based and view-based approaches. Point-based methods first voxelize the point cloud and input it into three-dimensional convolutional networks [1,2,3]. Other studies directly input unordered point cloud data into neural network models. These methods employ combinations of multi-layer perceptrons and max-pooling layers to capture multi-scale point cloud features, enabling feature extraction from local to global levels [4,5,6,7,8]. However, point-based methods generally involve high overall computational cost.

View-based methods project point clouds into multi-view two-dimensional images and then extract features using two-dimensional networks [9,10,11,12]. These methods simulate camera viewpoints, projecting the point cloud onto a 2D plane. The generated images are then processed by 2D networks for feature extraction and fusion, thereby improving object classification accuracy. Nevertheless, such classification models still rely on large datasets for pretraining to achieve satisfactory performance [13].

Vision–language pretrained models provide a new technical route for zero-shot point cloud recognition. Contrastive Language-Image Pre-training (CLIP) [14] learns transferable visual representations by aligning images and natural language descriptions in a shared embedding space, enabling cross-modal object classification [15,16]. PointCLIP [17] projects point clouds into multi-view depth maps and uses a frozen CLIP model for zero-shot classification. PointCLIP v2 [18] further introduces GPT-3 [19] to generate 3D semantic prompts. It also adopts voxelized projection to improve the quality of depth maps. MV-CLIP [20] improves the confidence of multi-view matching through view selection and hierarchical prompts. MVF-PointCLIP [21] further focuses on noisy views and covariance differences among views. DiffCLIP [22] combines Stable Diffusion with ControlNet to reduce the domain gap in the visual branch and improve zero-shot recognition performance. However, existing methods still mainly rely on contour-level projection. The representation of complex surfaces, local curvature, and depth hierarchy remains insufficient. This increases the domain gap between projected point cloud images and the natural images used for CLIP pretraining, which may reduce classification accuracy.

To address these limitations, this paper proposes a zero-shot 3D point cloud recognition framework based on graph-structured local geometric features and depth-aware multi-view projection. The framework builds upon PointCLIP v2. First, point clouds are modeled as a graph structure. Multi-scale neighborhoods are used to construct local graphs, and local covariance matrices are employed to extract geometric descriptors such as curvature, planarity, linearity, and scattering. This process captures the local surface shape and spatial structure of the point cloud. Next, a multi-view depth mapping mechanism is designed. Point cloud features are projected from multiple predefined viewpoints, and features along the depth direction are accumulated. For each pixel, point features from different depth layers are integrated using opacity-weighted volumetric rendering, generating multi-view two-dimensional feature maps with spatial visibility constraints. Finally, the multi-view images are input into a frozen CLIP model and aligned with 3D semantic text prompts to achieve zero-shot classification without additional training. This approach enhances the ability of two-dimensional projections to represent three-dimensional structural information and improves the alignment between multi-view features and the CLIP visual space.

The main contributions of this paper are summarized as follows:

1. A GNN-enhanced zero-shot 3D point cloud classification framework is proposed. The framework integrates multi-scale local geometric modeling, multi-view depth mapping, and frozen CLIP inference. It achieves zero-shot classification without training an additional 3D classification network.
2. A multi-scale local geometric feature extraction module is designed. This module represents point clouds as graph structures and computes covariance matrices within neighborhoods of different radii. Geometric descriptors, including curvature, planarity, linearity, and scattering, are then extracted through eigenvalue decomposition. This design enhances the representation of edges, curved surfaces, and complex surface regions.
3. A multi-view mapping mechanism based on depth-wise feature accumulation is proposed. This mechanism projects point cloud features from multiple viewpoints. It then performs opacity-weighted accumulation for different depth layers at the same pixel location. Compared with direct minimum-depth projection, this method preserves more depth hierarchy information and visibility relationships.
4. Experiments are conducted on ModelNet10, ModelNet40, and ScanObjectNN to evaluate the proposed method. The results show that the proposed method outperforms existing state-of-the-art methods in zero-shot classification.

## 2. Related Work

A common strategy for zero-shot 3D point cloud classification is to project 3D point clouds into 2D views. This allows visual priors learned by 2D pretrained models to be transferred to the 3D point cloud domain. In this process, modality bridging and prompt tuning are key to improving the generalization ability of zero-shot classification.

### 2.1. Modality Bridging of 3D Data Based on Voxel Grids

In the PointCLIP v2 model, ten predefined base viewpoints are generated by adjusting Euler angles and translation vectors to simulate camera viewpoints. As shown in Equations (1)–(3), each element sets the Euler angles (α, β, γ), corresponding to rotations around the X, Y, and Z axes, respectively.

(1) $$R_{X}\left(\alpha \right)=\left[\begin{array}{ccc} 1 & 0 & 0\\ 0 & cos\alpha & -sin\alpha \\ 0 & sin\alpha & cos\alpha \end{array}\right],$$

(2) $$R_{Y}\left(\beta \right)=\left[\begin{array}{ccc} cos\beta & 0 & sin\beta \\ 0 & 1 & 0\\ -sin\beta & 0 & cos\beta \end{array}\right],$$

(3) $$R_{Z}\left(\gamma \right)=\left[\begin{array}{ccc} cos\gamma & -sin\gamma & 0\\ sin\gamma & cos\gamma & 0\\ 0 & 0 & 1 \end{array}\right].$$

The rotation matrix is defined as $RM=R_{X}\left(\alpha \right)\cdot R_{Y}\left(\beta \right)\cdot R_{Z}\left(\gamma \right)$. Each element is assigned a translation vector $T=(t_{x},\;t_{y},t_{z})$, corresponding to translations along the X, Y, and Z axes, respectively. For any point *P* in the point cloud with coordinates (x, y, z), the coordinates of PPP after rotation and translation are denoted as *P*′:

(4) $$P^{\prime }=RM\cdot P+T,$$

(5) $$T={{[t}_{x},t_{y},t_{z}]}^{T}.$$

**Figure 1:**
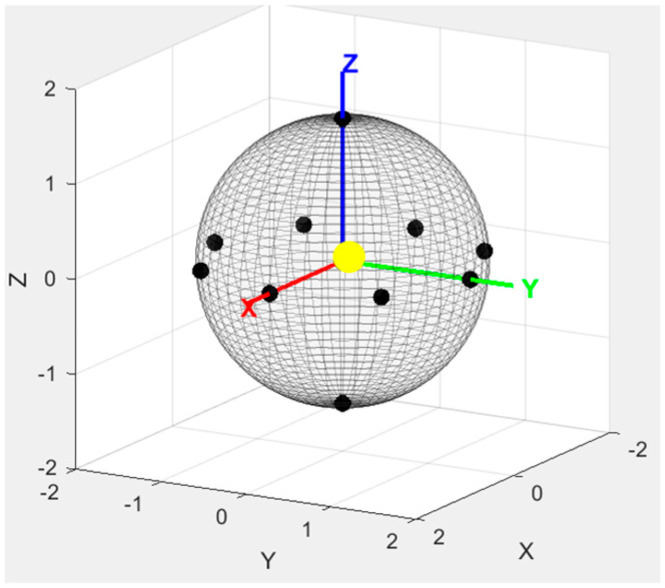
The figure shows the ten predefined projection viewpoints. The black dots represent the projection angles, and the yellow dot represents the point cloud object.

To provide an intuitive representation of the observation points for multi-view projection, ten viewpoint positions are marked in the 3D coordinate system, as shown in Figure 1. The point cloud object is placed at the center of the sphere, and the marked viewpoints are used to simulate camera viewpoints.

In the depth dimension, the minimum depth value is used as the pixel intensity for that coordinate. This voxel-grid-based modality bridging strategy aims to generate smooth and coherent two-dimensional images. However, this method primarily preserves the overall contour information and tends to overlook the local geometric details of the point cloud.

### 2.2. Prompt Learning in Vision

Prompt-based strategies enable cross-modal matching by generating category-specific textual descriptions [23,24]. CaFo et al. [25] converted semantic attributes of target objects into text prompts. Their method achieved strong performance in zero-shot object classification on various 2D datasets [25]. The text encoder in PointCLIP v2 [18] adopts a visual prompt tuning strategy. It generates text prompts with 3D-aware descriptions for different objects, thereby improving visual-language alignment accuracy. MV-CLIP [20] further introduces a hierarchical prompt strategy. It first uses category-level descriptions and then generates more refined appearance-related prompts for candidate classes. This improves zero-shot classification accuracy. Therefore, prompt tuning improves the generalization ability of zero-shot classification to unseen categories. It also plays a key role in multi-view feature fusion.

## 3. Methods

**Figure 2:**
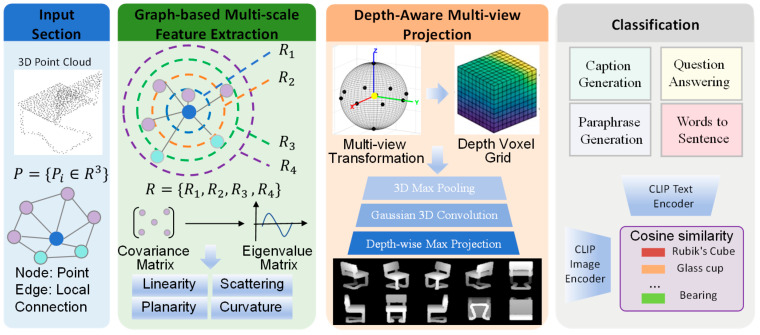
Overall framework of the proposed algorithm.

To address sparsity, the unordered nature of point clouds, local geometric information loss, and insufficient depth hierarchy in 2D projection, this paper proposes a zero-shot 3D object classification framework based on graph-structured local geometric features and depth-aware multi-view projection. The overall framework is shown in Figure 2.

In this framework, the 3D point cloud is first modeled as a graph structure. Points are treated as graph nodes, and spatial neighborhood relationships are treated as edges. Then, two-dimensional images are generated through graph-based multi-scale feature extraction, multi-view depth mapping, and depth-wise feature accumulation. These images are fed into the frozen CLIP image encoder and matched with text prompts for zero-shot classification.

### 3.1. Multi-Scale Local Geometric Feature Extraction Module

Point cloud data consists of discrete samples in three-dimensional space and is inherently unordered and irregular. Unlike two-dimensional images, point clouds do not have a fixed grid, and standard convolution cannot directly model local structures. To accurately describe the relationship between individual points and the overall structure, this paper employs a graph neural network as the backbone for feature extraction. Each point $=\{P_{i}\in R^{3}|i=1,2,\ldots N\}$ in the point cloud corresponds to a Node in the graph structure, where *N* is the number of points. Spatial adjacency relationships between points are represented as Edges in the graph. Therefore, the point cloud is modeled as a graph G=(Node,Edge).

The role of the graph neural network is not to independently map each point to a global representation. Instead, it models the relative geometric variations between a central point and its neighboring points through edge relationships. In this way, the representation of each point can integrate local contextual information. Accordingly, the relationship among “points, adjacency relationships, and local features” in a point cloud corresponds to “nodes, edges, and neighborhood aggregation” in a graph neural network. This structural consistency enables the Graph Neural Network (GNN) t to better preserve local structural features in unordered point cloud data.

First, the Euclidean distance is used to define the spatial distance between two points:

(6) $$d_{ij}={\left||P_{i}-P_{j}|\right|}_{2},$$

where $d_{ij}\;$ denotes the Euclidean distance between the i-th and j-th points in space. ${\left||\cdot |\right|}_{2}$ represents the L2 norm. Based on this distance, local neighborhood features around each central point are constructed. Considering that point cloud density varies with geometric shape, a single-scale neighborhood cannot capture all details. Therefore, a multi-scale radius set is introduced:

(7) $$R=\left\{R_{1},R_{2},\ldots ,R_{s}\right\},$$

where *R* denotes the set of radii across all scales, and $R_{s}$ denotes the neighborhood radius at the s-th scale. PointNet++ [5] has demonstrated that multi-scale grouping (MSG) based on ball query radii can mitigate the effects of non-uniform sampling density. CurveNet [26] incorporates an additional neighborhood feature extraction layer with a radius of 0.05 to model local surface variations more meticulously. Accordingly, the set of radii adopted in this work is R={0.05,0.1,0.2,0.4}. These radii progressively expand the receptive field. This enables the network to capture geometric information ranging from fine-grained local details to a broader structural context. Then, the local neighborhood of the i-th central point at the s-th radius scale is defined as

(8) $$N_{i,s}=\left\{j|{\left|\left|P_{i}-P_{j}\right|\right|}_{2}\leq R_{s}\right\},$$

where *j* denotes the index of a neighboring point that satisfies the radius constraint. A smaller radius scale focuses on fine-grained geometric structures around the central point, such as local protrusions and edge features. A larger radius scale covers a broader spatial region and describes the global contour characteristics of large point cloud regions and the spatial continuity between adjacent regions.

In the layer-wise feature extraction, the initial layer features are defined as

(9) $$h_{i}^{0}=p_{i},$$

where $h_{i}^{0}$ denotes the initial input feature of the i-th point, namely its three-dimensional coordinates. The feature of the i-th point at the l-th layer is denoted as $h_{i,l}$. Then, the relative feature between the central point and its neighboring point is defined as

(10) $$∆h_{i,j}^{l}=h_{i}^{l}-h_{j}^{l},$$

where $∆h_{i,j}^{l}$ denotes the feature offset of the neighboring point j relative to the central point *i*. To further characterize the geometric distribution of the local neighborhood, a local covariance matrix is computed at each radius scale. The neighborhood mean of the i-th point at the s-th scale is defined as

(11) $$\mu_{i}^{s}=\frac{1}{N_{i}^{s}}\cdot sum\left\{j\in N_{i}^{s}\right\}P_{j},$$

where $N_{i}^{s}$ denotes the number of points in the neighborhood at this scale. The set of neighboring points at each scale is then used to compute the local covariance matrix:

(12) $$C_{i}^{s}=\frac{1}{N_{i}^{s}}\cdot sum\left\{j\in N_{i}^{s}\right\}\left(P_{j}-\mu_{i}^{s}\right){\left(P_{j}-\mu_{i}^{s}\right)}^{T}$$

where $C_{i}^{s}$ denotes the local covariance matrix of the i-th point at the radius scale *s*. It is used to describe the distribution direction and dispersion of neighboring points in three-dimensional space. Eigenvalue decomposition is then performed on $C_{i}^{s}$, yielding three non-negative eigenvalues:

(13) $$\lambda_{i,1}^{s}\geq \lambda_{i,2}^{s}\geq \lambda_{i,3}^{s}\geq 0,$$

where $\lambda_{i,1}^{s}$, $\lambda_{i,2}^{s}$, and $\lambda_{i,3}^{s}$ denote the variance magnitudes along the three principal directions of the neighboring point set, respectively. When the largest eigenvalue is significantly larger than the other eigenvalues, the neighborhood is closer to a linear or edge-like structure. When $\lambda_{i,1}^{s}$ and $\lambda_{i,2}^{s}$ are large while $\lambda_{i,3}^{s}$ is small, the neighborhood is closer to a planar structure. When the three eigenvalues are relatively close to each other, the neighboring points are closer to a smooth volumetric structure. Therefore, local curvature, boundaries, and surface variations are not directly derived from distance alone. Instead, their geometric basis is provided by the eigenvalue distribution of the neighborhood covariance matrix.

The Euclidean distance is effective for constructing radius-constrained neighborhoods because it directly measures the spatial proximity between points. However, it treats all spatial directions equally and does not consider the local covariance structure of the point distribution. Therefore, this work introduces Mahalanobis distance [21,27]. It quantifies variations in the local geometric structure by calculating the eigenvalues of the local covariance matrix. This distance primarily measures the degree of deviation between the center point and its neighborhood in terms of local principal directions and variance.

To incorporate this local distribution information into point-wise distances, this paper further defines the Mahalanobis distance within the neighborhood radius *s*:

(14) $$D_{i,j}^{s}=sqrt\left({\left(P_{i}-P_{j}\right)}^{T}{\left(C_{i}^{s}+\varepsilon I\right)}^{-1}\left(P_{i}-P_{j}\right)\right),$$

where $D_{i,j}^{s}$ denotes the Mahalanobis distance between the i-th point and its neighboring point j at the radius scale *s*. ε is a small positive value used to avoid matrix singularity. I denotes the identity matrix. The radius-based Mahalanobis distance can capture boundary, abrupt-change, and non-smooth features within the local neighborhood.

To further aggregate local features, this paper constructs the edge features at the l-th layer and s-th scale as follows:

(15) $$e_{i,j}^{l,s}=concat\left(h_{i}^{l},h_{i}^{l}-h_{j}^{l},D_{i,j}^{s},G_{i,j}^{s}\right),$$

where $e_{i,j}^{l,s}$ denotes the edge feature between the central point *i* and its neighboring point *j*. The operation concat represents feature concatenation. $G_{i,j}^{s}$ represents the local geometric operator computed from the covariance features, which is defined as

(16) $$G_{i,j}^{s}=concat\left(L_{i}^{s},p_{i}^{s},S_{i}^{s},K_{i}^{s}\right),$$

$L_{i}^{s},p_{i}^{s},S_{i}^{s},K_{i}^{s}$ denote the linearity, planarity, scattering, and curvature variation of the neighborhood at the s-th radius scale, respectively. They are defined as follows:

(17) $$L_{i}^{s}={(\lambda }_{i,1}^{s}-\lambda_{i,2}^{s})/\lambda_{i,1}^{s},\;p_{i}^{s}={(\lambda }_{i,2}^{s}-\lambda_{i,3}^{s})/\lambda_{i,1}^{s},\;S_{i}^{s}=\lambda_{i,3}^{s}/\lambda_{i,1}^{s},\;K_{i}^{s}=\lambda_{i,3}^{s}/(\lambda_{i,1}^{s}+\lambda_{i,2}^{s}+\lambda_{i,3}^{s}).$$

By introducing these geometric descriptors into the edge features, the geometric representation of the point cloud no longer relies only on coordinate differences between points. Instead, it explicitly incorporates local structural distribution information.

The aggregated feature at each radius scale s can be represented as

(18) $$g_{i}^{l,s}=\max\left\{MLP\left(e_{i,j}^{l,s}\right)\right\}.$$

By aggregating neighborhood features across different radius scales, the feature at the l-th layer can be obtained as

(19) $$h_{i}^{l+1}=W_{l}\cdot concat\left(g_{i}^{l,1}+g_{i}^{l,2}+\ldots +g_{i}^{l,4}\right)+b_{l},$$

where $W_{l}$ and $b_{l}$ are the learnable weights and biases for scale fusion at the l-th layer. The feature $h_{i}^{l+1}$ of each point is updated by incorporating the local geometric responses from all four radius scales. This design allows the point features to capture both fine-grained local information and large-scale structural context, providing rich spatial semantics for downstream multi-view mapping.

**Figure 3:**
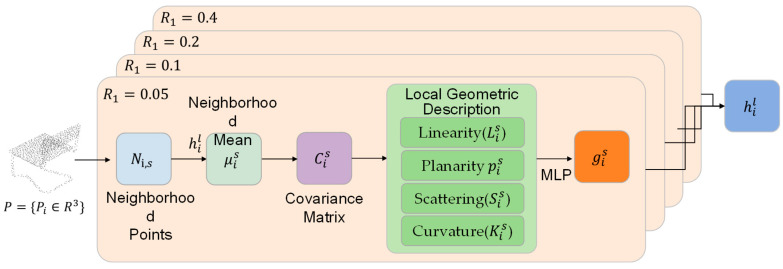
Overall framework of the Multi-Scale Local Geometric Feature Extraction Module.

The overall process of the multi-scale local geometric feature extraction module is shown in Figure 3.

### 3.2. Depth-Aware Multi-View Mapping

In the previous section, multi-level features are obtained through multi-scale neighborhood aggregation. These features contain the local geometric context of each point, neighborhood aggregation information, and representations that can be used for depth rendering. This paper further maps the 3D point cloud features into multi-view two-dimensional representations. These representations provide input for the frozen CLIP image encoder.

#### 3.2.1. Multi-View Rigid Transformation

To generate two-dimensional projections from *V* viewpoints, a rigid transformation is applied to each point $P_{i}={\left[x_{i},y_{i},z_{i}\right]}^{T}$:

(20) $$P_{i}^{v}=R_{v}P_{i}+t_{v},v=1,\ldots ,V,$$

where $P_{i}^{V}$ denotes the 3D coordinates of the i-th point under the v-th viewpoint. $R_{v}$ is the rotation matrix for the v-th viewpoint, and $t_{v}$ is the translation vector. In this paper, *V* is set to 10 viewpoints. By applying the rigid transformation, each point is mapped to the coordinate system of different viewpoints through the rotation matrix and translation vector, enabling multi-angle projection.

#### 3.2.2. Point-to-Image Projection

The point coordinates under each viewpoint are first normalized before being mapped to the voxel grid:

(21) $${\hat{P}}_{i}^{v}=\frac{P_{i}^{v}-c_{v}}{s_{v}},$$

where $c_{v}$ denotes the center of the point cloud under the v-th viewpoint, and $s_{v}$ denotes the maximum spatial scale of the point cloud, used for normalization. ${\hat{P}}_{i}^{v}={\left[{\hat{x}}_{i}^{v},{\hat{y}}_{i}^{v},{\hat{z}}_{i}^{v}\right]}^{T}$ represents the normalized point coordinates. The normalized coordinates are then mapped onto the image plane of size H × W:

(22) $$u_{i}^{v}=\frac{{\hat{x}}_{i}^{v}+1}{2}\cdot \left(W-1\right),$$

(23) $$q_{i}^{v}=\frac{{\hat{y}}_{i}^{v}+1}{2}\cdot \left(H-1\right),$$

where ($u_{i}^{v}$, $q_{i}^{v}$) are the pixel indices, representing the position of the i-th point on the 2D plane. (${\hat{x}}_{i}^{v},{\hat{y}}_{i}^{v}$) are the normalized x and y components. This step linearly scales the normalized coordinates to the image size range. This step aligns the image center with the point cloud origin and preserves the spatial scale of the projected view.

#### 3.2.3. Feature Accumulation and Volumetric Rendering Based on Depth Projection

On the 2D projection plane, each pixel may correspond to multiple point features at different depths. These features are accumulated using opacity-weighted summation. Volumetric integration is then performed along the depth direction to generate a 2D feature map. This method preserves depth hierarchy and visibility information. It also provides rich contextual information for multi-view 2D features. Let the feature of each pixel ($u_{i}^{v}$, $q_{i}^{v}$) in the 2D image be $F_{u,q}^{v}$, and let the corresponding opacity be $A_{u,q}^{v}$. The accumulated feature of this pixel is obtained by a weighted summation over all points projected onto the same pixel:

(24) $$F_{u,q}^{v}=\sum_{i\in S(u,q)}\alpha_{i}h_{i}^{l+1},$$

(25) $$A_{u,q}^{v}=\sum_{i\in S(u,q)}\alpha_{i},$$

where S(u,q) denotes the set of points projected onto the pixel (u, q). $\alpha_{i}$ denotes the opacity of the i-th point, which is determined by its depth along the z-axis. Initially, the depths within the same pixel are normalized as follows:

(26) $${\bar{z}}_{i}^{v}=\frac{z_{i}^{v}-z_{min}^{v}\left(u,q\right)}{z_{max}^{v}\left(u,q\right)-z_{min}^{v}\left(u,q\right)+\varepsilon },$$

where $z_{max}^{v}\left(u,q\right)$ and $z_{min}^{v}\left(u,q\right)$ denote the maximum and minimum depths, respectively, of all points projected onto the pixel (u, q). ε is a small constant introduced to prevent division by 0. To assign higher visibility to points closer to the viewpoint, we employ a depth attenuation function to compute the opacity:

(27) $$\alpha_{i}^{v}=\exp\left(-\beta {\bar{z}}_{i}^{v}\right),$$

where β is a hyperparameter that controls the intensity of depth attenuation.

Then, weighted integration is performed on the point features along the depth direction. This process considers the effect of the opacity of foreground points on ray propagation:

(28) $$T^{v}\left(k\right)={∐}_{k=1}^{K}\left(1-A^{v}\left(k\right)\right),$$

(29) $$I_{u,q}^{v}=\sum_{k=1}^{K}T^{v}\left(k\right)A^{v}\left(k\right)F^{v}\left(k\right),$$

where *K* denotes the number of depth layers. $T^{v}\left(k\right)$ denotes the ray transmittance before the k-th layer. $I_{u,q}^{v}$ denotes the generated 2D feature image under the v-th viewpoint. This continuous mathematical formulation is used to guide volumetric rendering integration. This formulation preserves depth-aware visibility information and incorporates local aggregated point features into the generated multi-view 2D maps.

The output multi-view feature set is defined as

(30) $$I=\left\{I^{1},I^{2},\ldots {,I}^{V}\right\}.$$

Through this mapping strategy, the GNN features of the point cloud are naturally transformed from the 3D space into 2D images. At the same time, local geometric information and visibility-aware weighting are preserved. This provides reliable inputs for cross-modal matching.

**Figure 4:**
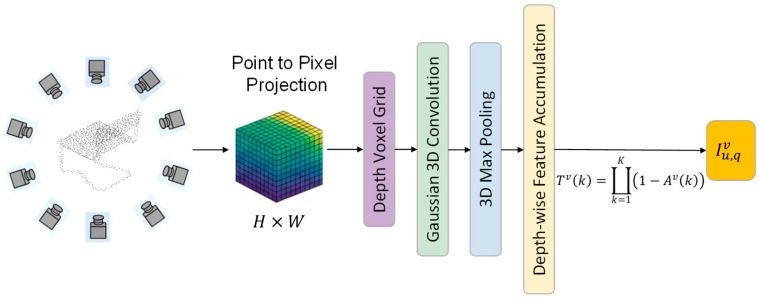
Overall framework of the Depth-Aware Multi-View Mapping Module.

The overall process of the Depth-Aware Multi-View Mapping module is shown in Figure 4.

### 3.3. Generation of 3D Semantic Text Prompts and Visual-Language Feature Alignment

**Figure 5:**
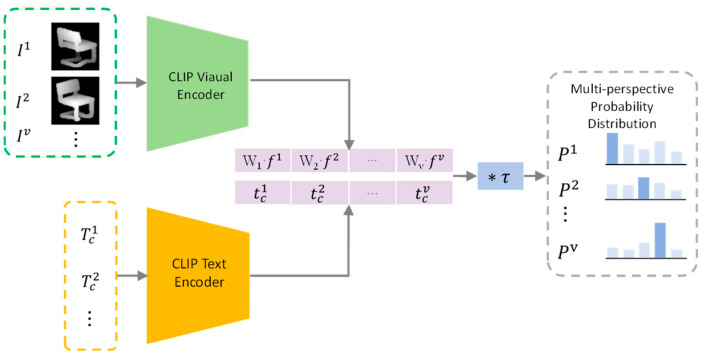
Visual-language feature alignment module.

In Section 3.2, the multi-view 2D feature maps I have been generated. These images preserve the spatial structure, edge information, and curvature information of the point cloud. Structured 2D feature maps are obtained through multi-view depth mapping and depth-wise feature accumulation. To achieve zero-shot recognition, the multi-view image features are aligned with category text prompts in a cross-modal space. The alignment process is shown in Figure 5.

Each category corresponds to a text prompt $T_{c}$, which describes 3D-aware attributes of the category. The text prompt is fed into the frozen CLIP text encoder to extract the text feature:

(31) $$t_{c}=CLIP\_Text\_Encoder\left(T_{c}\right).$$

The multi-view 2D feature maps I are extracted as visual features through the frozen CLIP image encoder:

(32) $$f^{v}=CLIP\_Image\_Encoder\left(I^{V}\right).$$

The zero-shot classification logits are computed using the dot product between visual and text features, and are then multiplied by the CLIP logit scale τ:

(33) $${logits}_{c}=\tau \cdot dot\left(t_{c},f^{v}\right),$$

where dot(,) denotes the dot product operation. Finally, the predicted category of the sample is obtained by selecting the class with the maximum logit value:

(34) $$y_{pred}={argmax}_{c}{logits}_{c}.$$

## 4. Experiments

### 4.1. Experimental Settings and Datasets

All experiments are conducted on a Linux platform using the PyTorch 2.1.2 framework. The software environment includes Python 3.8, Ubuntu 18.04, and CUDA 10.1. The experiments are performed on an NVIDIA RTX 3080 Ti GPU. The CLIP image encoder uses ViT-B/16 as the visual backbone. During the experiments, both the CLIP image encoder and the CLIP text encoder are kept frozen. No additional 3D data pretraining is performed.

The experiments are conducted on ModelNet10 [28], ModelNet40 [28] and ScanObjectNN [29]. ScanObjectNN is a real-world scene dataset. Compared with the ModelNet series, its point clouds contain occlusion, background noise, and pose perturbations. This paper evaluates the proposed method on three commonly used variants of ScanObjectNN: OBJ_ONLY, OBJ_BG, and PB_T50_RS. OBJ_ONLY contains object-only point clouds without background noise. OBJ_BG includes background context. PB_T50_RS introduces translation, rotation, and scaling perturbations.

For each input point cloud, the points are uniformly sampled and normalized before feature extraction. The point cloud input to the network contains 1024 points. During the projection stage, the point cloud is transformed into 10 predefined views using fixed rotation and translation parameters. The normalized point coordinates are then mapped into a voxel grid with an image resolution of 112 × 112 and 8 depth layers. These settings follow the voxel-based projection design of PointCLIP V2, providing a balance between projection detail and computational efficiency.

To achieve cross-modal alignment, we adopt CLIP ViT-B/16 as the visual-language backbone. During inference, both the visual encoder and text encoder are kept frozen. The prompts are generated based on four types of heuristic strategies: Caption Generation, Question Answering, Paraphrase Generation, and Keyword-to-Sentence Generation. By generating category-specific prompts containing depth-related semantic descriptions, the textual representations become more suitable for aligning with the 2D depth images generated from 3D point clouds.

### 4.2. Comparative Experiments

To comprehensively evaluate the effectiveness of the proposed zero-shot 3D object classification network, experiments were conducted on three public datasets. The classification results are presented in Table 1.

As shown in Table 1, our method outperforms the baseline model PointCLIP v2 across all evaluation tasks. Specifically, on ModelNet40, it achieves a classification accuracy of 67.21%, representing a 6.07% improvement over PointCLIP v2.

Methods such as GG3D and ReCon require pretraining. While ReCon exhibits stable performance, it still depends on large-scale labeled data and pretraining computations. In contrast, our method injects multi-radius, covariance-aware geometric descriptors during the 2D projection stage to generate high-fidelity 2D images, fully leveraging CLIP’s prior knowledge in the 2D domain. As a result, our approach does not rely on additional 3D pretraining, reducing both annotation costs and training overhead.

From the dataset perspective, the network shows improvements on both ModelNet10 and ModelNet40. This is mainly because these datasets contain regular geometric shapes and lack complex background interference. On the ScanObjectNN dataset, our method demonstrates robust performance. Under the OBJ_ONLY setting, the model achieves 53.18% accuracy, an improvement of 5.16% over PointCLIP v2. Under the OBJ_BG setting, where complex scene background noise is introduced, our method maintains high robustness, achieving 46.20% accuracy, which is 6.10% higher than PointCLIP v2.

This strong resistance to interference is mainly attributed to the multi-scale local geometric feature extraction module. By computing local geometric descriptors within decoupled Euclidean radii, the GNN implicitly learns to distinguish semantic object boundaries from unordered background noise. This helps reduce the interference of out-of-distribution (OOD) distortions in projected images on CLIP’s visual branch.

### 4.3. Ablation Experiments

To evaluate the contribution of each module to zero-shot point cloud classification performance, ablation experiments were conducted on the multi-scale local geometric feature extraction module (MLGE) and the depth-aware multi-view projection module (DMP). Table 2 shows the zero-shot classification accuracy of each module individually and in combination across the three datasets.

As shown in Table 2, both modules provide performance improvements. When only the MLGE module is enabled, the model achieves accuracies of 76.3% and 64.71% on ModelNet10 and ModelNet40, respectively, representing improvements of approximately 4.8% and 3.6% over the baseline. This indicates that local geometric features extracted from multi-scale neighborhood covariance matrices effectively capture curvature, planarity, linearity, and scattering of the point cloud, thereby enhancing the representation of complex surfaces and local structures.

When only the DMP module is enabled, the model achieves accuracies of 75.32% and 63.2% on ModelNet10 and ModelNet40, respectively, representing improvements of approximately 3.84% and 2.06% over the baseline. This demonstrates that depth-wise feature accumulation preserves more depth hierarchy and visibility information during 2D projection. Compared with direct minimum-depth projection, this mechanism reduces structural loss caused by depth compression.

Within the ablation setting, enabling both the MLGE and DMP modules yields the best performance across all evaluated datasets. Accuracy reaches 80.5% on ModelNet10 and 67.21% on ModelNet40. For the ScanObjectNN variants, accuracies are 53.18%, 46.20%, and 37.71%, respectively. These results indicate that the two modules are complementary: These results indicate that the two modules are complementary: the multi-scale local geometric feature extraction module enhances local structural representation, whereas the depth-aware projection module improves depth-layer encoding in multi-view projections. Together, they generate 2D representations that are better suited for recognition by the CLIP image encoder.

**Figure 6:**
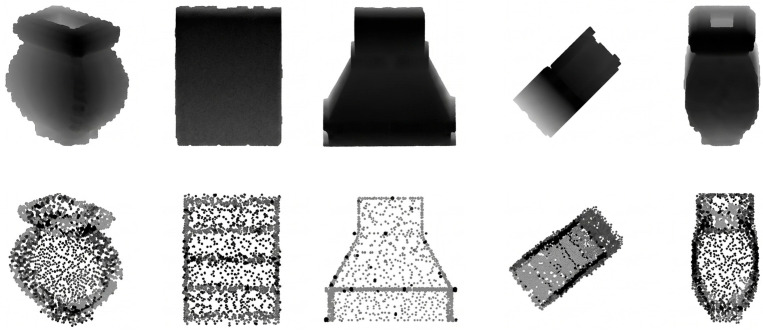
The first and second rows respectively show the projection results of the same point cloud under the same viewing angle, using pointCLIP v2 and the Depth-Aware projection module.

To further verify the effectiveness of the proposed depth-aware projection, we provide a qualitative comparison with the original PointCLIP V2 projection, as shown in Figure 6. The first row presents the projection results generated by the PointCLIP V2 baseline, while the second row shows the results produced by the proposed depth-aware projection. It can be observed that the baseline projection mainly retains a coarse global silhouette, but tends to lose fine-grained depth variation and local visibility cues due to the compression of multiple depth responses into a relatively limited representation. By contrast, the proposed method preserves richer structural information, including clearer object boundaries, more distinguishable local shape patterns, and more explicit depth-layer distributions. In particularly, for objects with non-trivial surface geometry, the proposed projection better maintains depth discontinuities and visibility relationships, which indicates that the depth-aware accumulation strategy provides a more informative 2D representation for subsequent feature extraction and classification. 

### 4.4. Visualization Analysis

**Figure 7:**
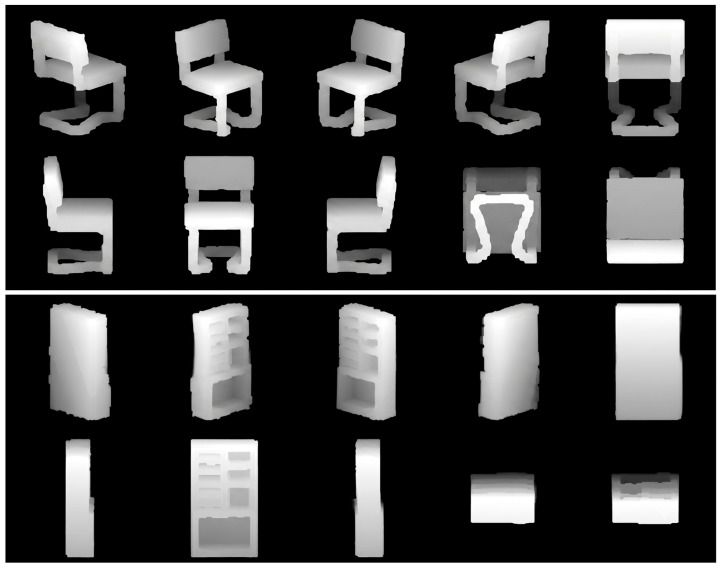
Visualization of multi-view depth-compressed 2D projections. Given an input 3D point cloud, the proposed method transforms the point cloud into multiple 2D representations from different viewpoints. Each image corresponds to one predefined viewing direction, preserving the overall object geometry, shape contours, and viewpoint-dependent structural information.

To visually evaluate the ability of the proposed method to preserve 3D structural information, intermediate feature maps of selected test samples are visualized. Figure 7 shows the visualization results after multi-scale local feature extraction and multi-view depth mapping.

As shown in Figure 7, object boundaries, edges, and local convex–concave features are clearly preserved in the 2D projections. This indicates that multi-view voxel grids retain key geometric information of the point cloud even during depth compression. Vertical and horizontal depth compression results reflect the object’s hierarchical structure. For example, for furniture objects, drawer levels, box partitions, and edge contours remain clearly distinguishable in the projected images. This demonstrates that depth-wise feature accumulation preserves essential spatial structure and visibility information while compressing 3D point clouds into 2D planes.

**Figure 8:**
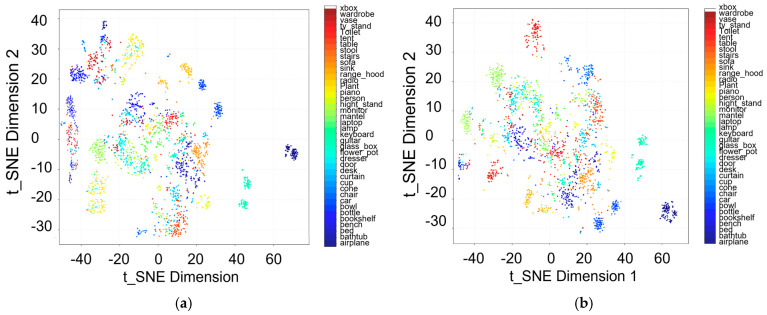
T-SNE visualization of encoded features for (**a**) PointCLIP v2 and (**b**) Proposed on the ModelNet40 test set. Each point represents an encoded feature sample, and different colors correspond to different object categories. Compared with PointCLIP V2, the proposed method produces more compact intra-class distributions and clearer inter-class separations, indicating improved discriminative feature representation.

Furthermore, due to the inherent sparsity of point clouds, local structures are expanded using max-pooling dilation and Gaussian convolution smoothing. This produces smoother and more continuous 2D images. As shown in Figure 8, the mapping process reduces discontinuities caused by sparse point clouds, resulting in more continuous 2D projections. This facilitates the frozen CLIP image encoder in extracting stable visual features.

To further evaluate the feature representation capabilities of the proposed model, we employed t-SNE (t-Distributed Stochastic Neighbor Embedding) for the downscaling and clustering analysis of encoder features. This technique was applied to the ModelNet40 test set, enabling a comparative visual analysis between proposed model and the widely used PointCLIP v2. The clustering results are presented in Figure 8a for PointCLIP v2 and Figure 8b for proposed model.

The PointCLIP v2 clustering has poor separability and unclear boundaries. In contrast, the feature space of proposed method, as visualized through t-SNE, demonstrates a substantial improvement in cluster separability. The clusters are independently distributed with minimal overlap, and most are tightly grouped.

To further verify the applicability of the proposed method to real-world collected data, point clouds of objects such as bicycles and plastic bottles were acquired using a PandarXT-16 LiDAR. The sensor has 16 scan channels and provides a 360° horizontal field of view and a 30° vertical field of view ranging from −15° to +15°. Its measurement range is 0.05–120 m. The ranging accuracy is 1 cm The LiDAR outputs distance, azimuth, and reflectivity measurements.

**Figure 9:**
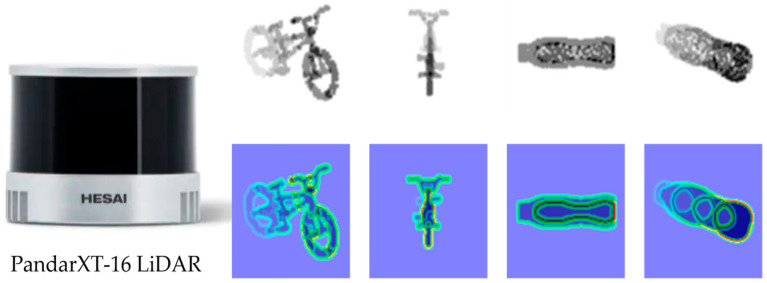
Visualization of depth-minimum projections and local geometric responses for real-world LiDAR point cloud samples. The upper row shows the original point cloud projections from different viewpoints, while the lower row presents the corresponding local geometric responses generated by the proposed feature extraction module. The highlighted regions indicate geometry-sensitive areas, including object boundaries and local surface structures.

The raw LiDAR data were decoded from UDP packets. Each data block contains an azimuth angle, and each laser channel provides a distance and reflectivity measurement. Based on the decoded distance, the azimuth angle, and the vertical angle of the corresponding laser channel, each laser return was converted into a 3D point in the LiDAR coordinate system. Multi-view projection visualization was then performed. The results are shown in Figure 9.

The first row shows the projection results obtained by directly using the minimum depth value. The projected point cloud shows a clearly sparse and discontinuous distribution on the 2D plane. The second row shows the explicit representation of geometric features after multi-scale local geometric modeling. This indicates that the multi-scale local geometric feature extraction module can effectively capture curvature, edges, and other object information. It not only preserves the overall spatial contour of the target but also enhances the distinguishability of local geometric details.

However, in this experiment, only a small amount of point cloud data of real-world objects was collected. The samples have limitations in terms of category diversity, collection distance, scene complexity, and the number of outliers.

### 4.5. Computational Complexity

To evaluate the computational efficiency of the proposed method, we analyze theoretical complexity and actual inference time. The main computational costs of the method arise from three steps: multi-scale neighborhood construction, local geometric feature extraction, and multi-view depth mapping. Let the input point cloud contain *N* points, with feature dimension *d*, *S* radius scales, *K* average neighboring points per scale, *V* viewpoints, and projection resolution R × R.

First, in the multi-scale neighborhood construction stage, if a global graph connection is used, the computational complexity of point-to-point relationships is O(N²d), which increases rapidly as the number of points grows. This paper adopts radius-constrained local neighborhood search, where each point is connected only to *K* neighbors. Therefore, in the local geometric feature extraction stage, covariance matrices are computed for each neighborhood at each scale, and geometric descriptors such as curvature, linearity, planarity, and scattering are extracted. The main complexity of this stage is still determined by neighborhood traversal and feature aggregation, resulting in an overall complexity of approximately O(SNKd).

In the multi-view depth mapping stage, the depth-wise feature accumulation depends on the projection resolution and the number of points along each pixel’s depth layers. The complexity is approximately $O(VR²K_{z})$, where $K_{z}$ denotes the average number of points accumulated along the depth direction for each pixel. Therefore, the overall complexity of the multi-view mapping stage is approximately $O(SNKd\;+\;VR²K_{z})$.

To reduce actual inference overhead, this paper employs parallel processing. Neighborhood computations at different radius scales are independent and can be processed in parallel. During depth mapping, feature accumulation at different pixel positions can also be performed in parallel. This approach increases local geometric modeling capacity while reducing overall inference time and memory usage.

To further verify practical efficiency, inference times of different methods are recorded on the ModelNet40 test set, and the results are presented in Table 3.

The inference latency of DiffCLIP is the longest because it relies on a computationally intensive and memory-demanding diffusion model to generate RGB images. The MVF-PointCLIP model incorporates an SFA module and a Mahalanobis Distance module, which also increases latency. In contrast, the proposed method employs parallel processing, reducing computational complexity while improving classification efficiency, resulting in lower latency than MVF-PointCLIP and much lower inference cost than DiffCLIP. These results indicate that the proposed method achieves a favorable efficiency–accuracy trade-off while maintaining zero-shot inference capability.

**Figure 10:**
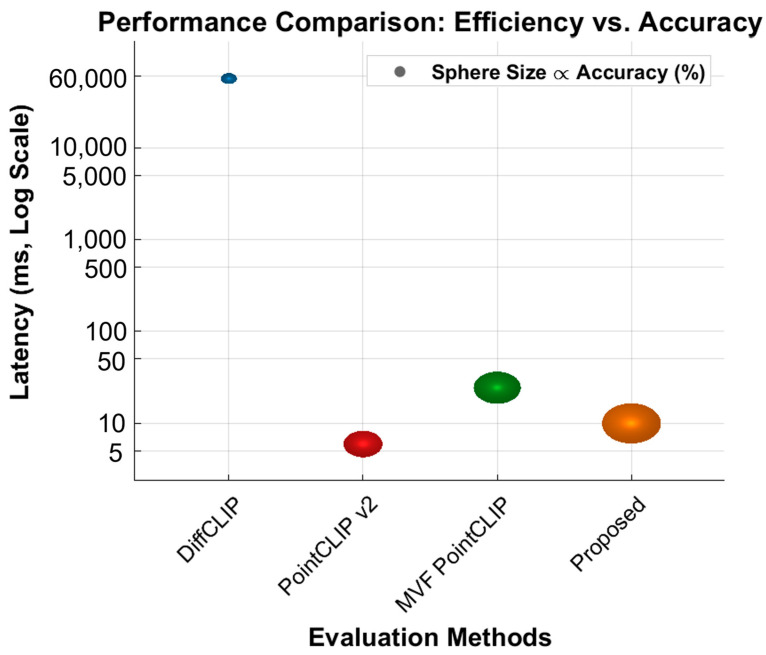
Efficiency–accuracy comparison of different methods.

Figure 10 illustrates the relationship between inference efficiency and classification accuracy for different methods. The proposed method achieves higher accuracy compared with PointCLIP v2, while significantly reducing inference overhead compared with DiffCLIP. Therefore, the proposed framework is better suited for zero-shot point cloud classification scenarios without additional 3D labeled data.

## 5. Conclusions

The proposed zero-shot 3D object classification framework, based on multi-scale local geometric feature extraction and depth-aware multi-view projection, is designed to address geometric detail loss in 2D projections generated from 3D point clouds. The point cloud is modeled as a graph structure, where multi-scale neighborhood graphs and local covariance matrices are used to extract geometric descriptors, enhancing the representation of local structures. Multi-view projections are then performed, and features are accumulated along the depth direction to generate 2D projected images. This process preserves 3D spatial hierarchy and visibility constraints, making the projections more suitable for stable feature extraction by the CLIP model.

Experimental analysis shows that the proposed method outperforms the baseline PointCLIP v2 and other existing approaches on ModelNet10, ModelNet40, and ScanObjectNN datasets. Visualization results further demonstrate that the combination of multi-scale geometric features and depth-wise accumulation preserves edges, curvature-related structures, and spatial hierarchy. In addition, parallel processing is employed to improve inference efficiency. Therefore, the proposed framework provides a practical solution for efficient zero-shot point cloud classification in open scenarios.

However, since CLIP is pretrained on color images, the distribution gap between grayscale and color images may affect its performance. In future work, we will explore geometry-aware rendering and generative models to further improve the fidelity of 2D projections and reduce the gap between projected images and natural images.

## Figures and Tables

**Table 1:** Zero-shot classification accuracy of various methods on ModelNet10, ModelNet40, and ScanObjectNN variants.

Method	2D Pre-Train	3D Pre-Train	ModelNet10	ModelNet40	OBJ_ONLY	OBJ_BG	PB_T50_RS
Cheraghian [30]	×	√	68.5	N/A	N/A	N/A	30.53
CLIP2Point [31]	√	√	66.63	49.38	35.46	30.46	23.32
PointMLP + GG3D [32]	×	√	64.1	50.4	N/A	N/A	25.0
PointTransformer + GG3D [32]	×	√	67.3	50.6	N/A	N/A	25.6
ReCon [33]	√	√	75.6	61.7	43.7	40.4	30.5
PointCLIP [17]	√	×	30.23	23.78	21.34	19.27	15.38
DiffCLIP [22]	√	×	82.4	54.2	45.3	43.2	35.2
MVF-PointCLIP [21]	√	×	75.99	63.65	53.01	47.68	35.57
PointCLIP v2 [18]	√	×	71.48	61.14	48.02	40.1	34.32
Proposed	√	×	80.5	67.21	53.18	46.2	37.71

**Table 2:** Ablation study of the multi-scale local geometric feature extraction module and the depth-aware projection module.

MLGE	DMP	ModelNet10	ModelNet40	OBJ_ONLY	OBJ_BG	PB_T50_RS
×	×	71.48	61.14	48.02	40.1	34.32
√	×	76.3	64.71	52.07	46.53	37.0
×	√	75.32	63.2	50.23	42.67	35.18
√	√	80.5	67.21	53.38	46.2	37.71

**Table 3:** Time consumption of zero-shot inference on ModelNet40.

Method	Latency	Test Inference Time
DiffCLIP [22]	56.96 s	862 min
PointCLIP v2 [18]	5.9 ms	53.84 s
MVF_PointCLIP [21]	24.3 ms	22.13 s
Proposed	9.92 ms	23.1 s

## Data Availability

The data supporting the findings of this study are available from the corresponding author upon reasonable request.
